# Prevalence of major diseases in common marmosets (*Callithrix jacchus*) at the Central Institute for Medicine and Life Science: a retrospective study

**DOI:** 10.3389/fvets.2025.1548757

**Published:** 2025-07-02

**Authors:** Takayuki Mineshige, Takashi Inoue, Terumi Yurimoto, Kenya Sato, Kenji Kawai, Erika Sasaki

**Affiliations:** ^1^Division of Advanced Physiology, Central Institute for Experimental Medicine and Life Science, Kawasaki, Japan; ^2^School of Veterinary Medicine, Azabu University, Sagamihara, Japan; ^3^Faculty of Veterinary Medicine, Okayama University of Science, Imabari, Japan; ^4^Pathology Center, Central Institute for Experimental Medicine and Life Science, Kawasaki, Japan; ^5^Keio University Regenerative Medicine Research Center, Kawasaki, Japan

**Keywords:** *Clostridioides difficile*, common marmoset, marmoset duodenal dilation syndrome, marmoset wasting syndrome, veterinary pathology

## Abstract

Common marmosets (*Callithrix jacchus*) are increasingly being used in neuroscience and biomedical research due to their small size, and ease of handling. Despite their growing research importance, marmoset colonies face health management challenges. Marmoset wasting syndrome (MWS), marmoset duodenal dilatation syndrome (DDS), and *Clostridioides difficile*-associated disease (CDAD) are the leading causes of mortality in marmosets. We retrospectively analyzed the necropsy records of 192 marmosets based on clinical and pathological criteria at the Central Institute for Medicine and Life Science between 2017 and 2020 to determine the incidence of major diseases and associated treatment modalities. MWS is prevalent in older animals and is characterized by progressive weight loss, hypoalbuminemia, and chronic enteritis. DDS, identified in younger marmosets, is associated with gastrointestinal distress and requires a specialized liquid diet and supportive care. CDAD, which is often triggered by antibiotic administration, leads to sudden death in approximately 68% of cases. This study underscores the need for tailored veterinary care, including early diagnosis, nutritional management, and cautious antibiotic use, to improve marmoset health and reduce mortality rates. Further research on the pathogenesis of these diseases, including gut microbiota analysis, histopathological examination, and diagnostic imaging, is essential for developing effective prevention and treatment strategies.

## 1 Introduction

The common marmoset (*Callithrix jacchus*), a New World primate, has been widely used in neuroscience and biomedical research in recent years. Marmosets have several advantages as experimental animals, including their small size (300–500 g), ease of handling, fecundity, and early sexual maturity. Recent progress in transgenic and genome editing technology has expanded research on marmosets (1–4).

As with other species, veterinary care is crucial for monitoring marmoset health and protecting marmoset colonies. Marmosets have been reported to develop conditions such as marmoset wasting syndrome (MWS) (3–7), renal disease (8, 9), small intestinal adenocarcinoma (4, 10, 11), and gastrointestinal tract lymphoma (4, 10, 12). Marmoset duodenal dilatation syndrome (DDS) (13–15) and *Clostridioides* (previously *Clostridium*) *difficile*-associated disease (CDAD) have been reported within the last decade (3, 4, 16, 17), but their pathogeneses remain unknown. These conditions are associated with substantial morbidity and mortality, with varying rates reported across institutions (18–20). Despite increasing recognition of these conditions, diagnostic consistency and treatment strategies remain poorly standardized, particularly for recently identified conditions like DDS and CDAD.

Given these challenges, long-established institutions with large marmoset colonies play a critical role in disease surveillance and management. The Central Institute for Medicine and Life Science (CIEM), formerly called the Central Institute for Experimental Animals (CIEA), has one of the largest marmoset colonies in the world (1–3, 13, 21–25), maintaining up to 800 marmosets. CIEM has extensive experience in marmoset care and research, including pioneering transgenic models (1) and genome-editing technology (2), and provides structured health and welfare management for these animals.

No study has comprehensively addressed these conditions within a single facility, particularly focusing on newly recognized but poorly understood entities, such as DDS and CDAD. Therefore, this study aimed to systematically characterize disease prevalence and clinicopathological features in a large captive marmoset colony at the CIEM. Through integrated analysis of clinical, hematological, pathological, and imaging findings, MWS, DDS, and CDAD emerged as the major causes of morbidity and mortality. By delineating disease patterns and evaluating current management strategies, this study provides practical insights to inform evidence-based veterinary care and improve colony health management.

## 2 Materials and methods

### 2.1 Animals

This study was performed in strict accordance with the Regulations for Animal Experimentation of CIEM, which are based on the Guidelines for Proper Conduct of Animal Experiments (Science Council of Japan, 2006). The animal experiment protocol was approved by the Institutional Animal Care and Use Committee of the CIEA (approval no. 16002A). Third-party verification has confirmed that appropriate animal testing is conducted scientifically while promoting voluntary management of such testing and considering animal welfare.

Animals were housed in appropriately sized cages with enrichment materials and maintained at 26–28°C, 40–60% humidity, under a 12 h:12 h light/dark cycle. The animals were fed a commercial New World primate diet (CMS-1 M; CLEA Japan Inc., Tokyo, Japan) with added ascorbic acid (Nacalai Tesque Inc., Kyoto, Japan), vitamins A, D3, and E (Duphasol AE3D; Kyoritsu Seiyaku Co., Ltd., Tokyo, Japan), honey, and tap water ad libitum. In addition to the normal diets, gum arabic, sponge cakes, biscuits, marshmallows, or apple jelly were fed to the animals. Animals were checked multiple times daily by experienced animal keepers and veterinarians and were weighed every month. The animals were tested annually using fecal samples and were negative for *Salmonella* spp., *Shigella* spp., and *Yersinia pseudotuberculosis*.

Retrospective analyses of wild-type marmosets were conducted by reviewing the necropsy records at the CIEM over the 3-year period from 2017 to 2020. The marmosets included in this study were either those that had been used in genetic engineering research or neurological studies or those that had not yet been subjected to experimental procedures. Marmosets younger than 6 months of age, those that were genome-edited or transgenic, and those that died as a direct result of experimental procedures were excluded from the analysis.

### 2.2 Humane endpoint and criteria

Clinical care was done by three veterinarians. Euthanasia was performed on animals under veterinary care when the animal’s recovery was unlikely due to severe physical conditions, such as weight loss of ≥ 30% compared to the baseline body weight (before the start of the experiment or onset of the disease), collapse, piloerection, or chronic diarrhea. The animals were deeply anesthetized with 50 mg/kg ketamine, 4 mg/kg xylazine, and 1–3% isoflurane and then euthanized by exsanguination from the heart or abdominal aorta due to their moribund condition.

### 2.3 Diagnostic criteria for diseases

#### 2.3.1 MWS

The modified diagnostic criteria for MWS were based on the published criteria proposed by Baxter, et al. (26). The following criteria were used for selection: (i) progressive weight loss of 0.05% of the peak body weight (BW) per day, estimated using monthly weight measurements, (ii) weight loss of >10% from peak BW, (iii) BW of < 300 g, and (iv) no evidence of other diseases, including marmoset DDS, sepsis, diabetes, infectious enteritis, or tumors.

#### 2.3.2 DDS

The diagnostic criteria for DDS was a maximum diameter of the descending duodenum of >12 mm, according to our previous report (13). Duodenal compression by the superior mesenteric artery, tumors, and intussusception were not observed at the inferior flexure. No dilation was evident in other parts of the duodenum, jejunum, or ileum.

#### 2.3.3 CDAD

CDAD was diagnosed based on the detection of *C. difficile* glutamate dehydrogenase (GDH) antigen or toxins in the fecal samples along suggestive clinical signs (16, 17). Fecal samples were tested using a rapid membrane enzyme immunoassay (C Diff Quick Chek Complete; Alere, Chiba, Japan) and confirmed to be positive for *C. difficile* glutamate dehydrogenase antigen and toxin A/B.

#### 2.3.4 Other diseases

Hemoperitoneum was diagnosed based on severe intra-abdominal bleeding and anemia. Renal failure was diagnosed based on a serum creatinine concentration > 1.0 mg/dL without other diseases. All neoplastic lesions in this study were diagnosed using histopathological analysis of postmortem tissues.

### 2.4 Blood tests

Blood samples were collected from the marmosets at the time of euthanasia or between diagnosis and death. Complete blood counts were analyzed using a Sysmex XT-2000i (Sysmex Corporation, Kobe, Japan), whereas clinical chemistry was assessed using a DRI-CHEM 7000 (Fujifilm Corporation, Tokyo, Japan). The analyses were performed based on the availability of stored serum samples; therefore, not all animals were included in the comparison. We calculated the corrected calcium using the following formula:


$$\begin{array}{l} \text{corrected calcium}\;(\mathrm{mg}/\mathrm{dL})=\text{measured}\;\mathrm{Ca}\;(\mathrm{mg}/\mathrm{dL})\\ -\text{serum albumin}\;(g/\mathrm{dL})+3.5 \end{array}$$

### 2.5 Pathological analysis

All necropsies were performed by the veterinarians. Bacterial cultures were conducted on suppurative bile samples collected from five diseased animals at the ICLAS Monitoring Center.

Histopathological examinations were performed on samples from 42 marmosets (Supplementary Table S1). All major organs were fixed in 10% neutral-buffered formalin, embedded in paraffin, cut, and stained with hematoxylin and eosin. Slides were examined by a veterinary pathologist.

### 2.6 Statistical analyses

All statistical analyses were performed using Python (ver. 3.12.3) with the Scipy library (ver. 1.11.3). Continuous variables such as age and BW are expressed as means ± standard deviation (SD). The normality of the data distribution was assessed using the Shapiro–Wilk test. Nonparametric statistical methods were used, as the age and BW data were not normally distributed (*p* < 0.05).

Differences between disease groups (MWS, DDS, CDAD, and all cases) were analyzed using the Kruskal–Wallis test. Pairwise comparisons were conducted using Dunn’s post-hoc test with Bonferroni correction to control for multiple comparisons when significant differences were identified. Statistical significance was denoted by *p* < 0.05.

For categorical variables such as mortality classification (death during husbandry vs. euthanasia), group differences were evaluated using the chi-squared test. Fisher’s exact test was used when the expected cell frequencies were < 5. The significance threshold for categorical variables was set at *p* < 0.05.

## 3 Results

During the study period, 192 marmosets were necropsied after excluding neonates, genetically modified animals, and research necropsies. The signalments of the necropsied marmosets included in the present study are listed in Supplementary Table S1. These animals included 107 males and 85 females, and their ages ranged 1.4–17.0 years (average: 8.1 ± 3.2 years). Of the 192 necropsied marmosets, 143 were euthanized. The remaining 49 died during care, including 33 cases under clinical treatment and 16 cases of sudden death (Supplementary Table S1).

Retrospective analysis of necropsy records revealed that MWS (*n* = 59), DDS (*n* = 41), and CDAD (*n* = 19) were the most common causes of mortality in marmosets at the CIEM, collectively accounting for 62.0% of all cases. Other causes of death included hemoperitoneum (*n* = 6), gastrointestinal diseases (excluding MWS and DDS) (*n* = 5), small intestinal adenocarcinoma (n = 5), esophageal dilation (*n* = 5), heart failure (*n* = 5), unknown causes (*n* = 10), and other diseases (*n* = 37) (Supplementary Table S1).

First, we examined the overall demographic patterns and causes of death; subsequently, we focused on the three major diseases—MWS, DDS, and CDAD—which together accounted for 62.0% of all cases. Detailed clinicopathological characteristics for each disease are presented below.

### 3.1 Pathological analysis

Histopathological examinations were performed on samples from 42 marmosets (Supplementary Table S1). The examined cases include 11 animals with MWS, 12 with DDS, 5 with small intestinal adenocarcinoma, 3 with lymphoma, 2 with CDAD, and several others with conditions, such as small intestinal ulcers, hemoperitoneum, and renal disease. The affected individuals consisted of 22 males and 20 females, with an average age at death of 8.5 ± 2.6 years and a mean body weight of 267.8 ± 48.8 g.

### 3.2 MWS

Among the identified diseases, MWS was the most prevalent and primarily affected older marmosets. The clinical, laboratory, and pathological features of the MWS cases are summarized below.

Fifty-nine necropsied marmosets were diagnosed with MWS, accounting for 28.3% of all the non-experimental deaths during this period. Their ages ranged 4.1–17.0 years, with an average of 9.7 ± 2.3 years. In addition, 51/59 (85.7%) animals were aged 6–12 years (Supplementary Table S2). The MWS group was significantly older than the DDS and CDAD groups (*p* < 0.001 for both comparisons). No sex predilection was observed (*p* = 1.0). The average BW at the time of death for the MWS group was 237 ± 32 g, which was significantly less than those of the other disease groups (*p* < 0.001).

Blood tests revealed hypoalbuminemia (< 3.0 g/dL) in 42 of 55 animals, elevated total bilirubin (> 0.5 mg/dL) in 34 of 54 animals, and anemia (Ht < 30%) in 26 of 49 animals (Table 1). The mean values were 2.5 ± 0.6 g/dL for albumin concentration, 0.9 ± 1.5 mg/dL for total bilirubin concentration, and 28.9 ± 7.5% for Ht (Supplementary Table S3). The sample sizes varied due to the availability of stored serum samples.

**Table 1:** Proportion of marmosets with abnormal blood test results at the CIEM.

Abnormality	Reference value	MWS^a^	DDS^b^	CDAD^c^	Total
Leukocytosis	WBC > 100 10^2/uL	3/51 (5.9%)	1/25 (4.0%)	1/6 (16.7%)	7/112 (6.2%)
Anemia	Ht < 30%	26/49 (53.1%)	10/25 (40.0%)	2/6 (33.3%)	51/110 (46.4%)
Hypoalbuminemia	ALB <3.0 g/dL	42/55 (76.4%)	13/32 (40.6%)	4/6 (66.7%)	75/132 (56.8%)
Elevated GPT	GPT > 10 U/L	21/54 (38.9%)	3/32 (9.4%)	1/5 (20.0%)	37/129 (28.7%)
Elevated GGT	GGT > 10 U/L	7/54 (13.0%)	9/32 (28.1%)	1/5 (20.0%)	29/130 (22.3%)
Hyperbilirubinemia	T-bil > 0.5 mg/dL	34/54 (63.0%)	15/32 (46.9%)	2/5 (40.0%)	68/129 (52.7%)
Elevated creatinine	CRE > 0.5 mg/dL	7/55 (12.7%)	5/32 (15.6%)	2/5 (40.0%)	30/130 (23.1%)
Hyperphosphatemia	IP > 5 mg/dL	20/55 (36.4%)	15/32 (46.9%)	6/6 (100.0%)	65/132 (49.2%)
Hypercalcemia	Corrected Ca (for Alb) > 11 mg/dL	4/54 (7.4%)	0/32 (0.0%)	0/6 (0.0%)	7/131 (5.3%)
Hypocalcemia	Corrected Ca (for Alb) < 8.5 mg/dL	18/55 (32.7%)	13/32 (40.6%)	13/32 (40.6%)	13/32 (40.6%)
Hyponatremia	Na < 135 mEq/L	2/49 (4.1%)	5/32 (15.6%)	3/7 (42.9%)	16/131 (12.2%)
Hypokalemia	K < 3.0 mEq/L	17/49 (34.7%)	10/32 (31.2%)	2/7 (28.6%)	47/131 (35.9%)
Hypochloremia	Cl < 95 mEq/L	0/49 (0.0%)	5/32 (15.6%)	3/7 (42.9%)	14/131 (10.7%)

No. of marmosets abnormal/measured (% abnormal); CIEM, Central Institute for Experimental Medicine and Life Science.

a Marmoset wasting syndrome.

b Marmoset duodenal dilatation syndrome.

c Clostridioides difficile-associated disease.

**Figure 1:**
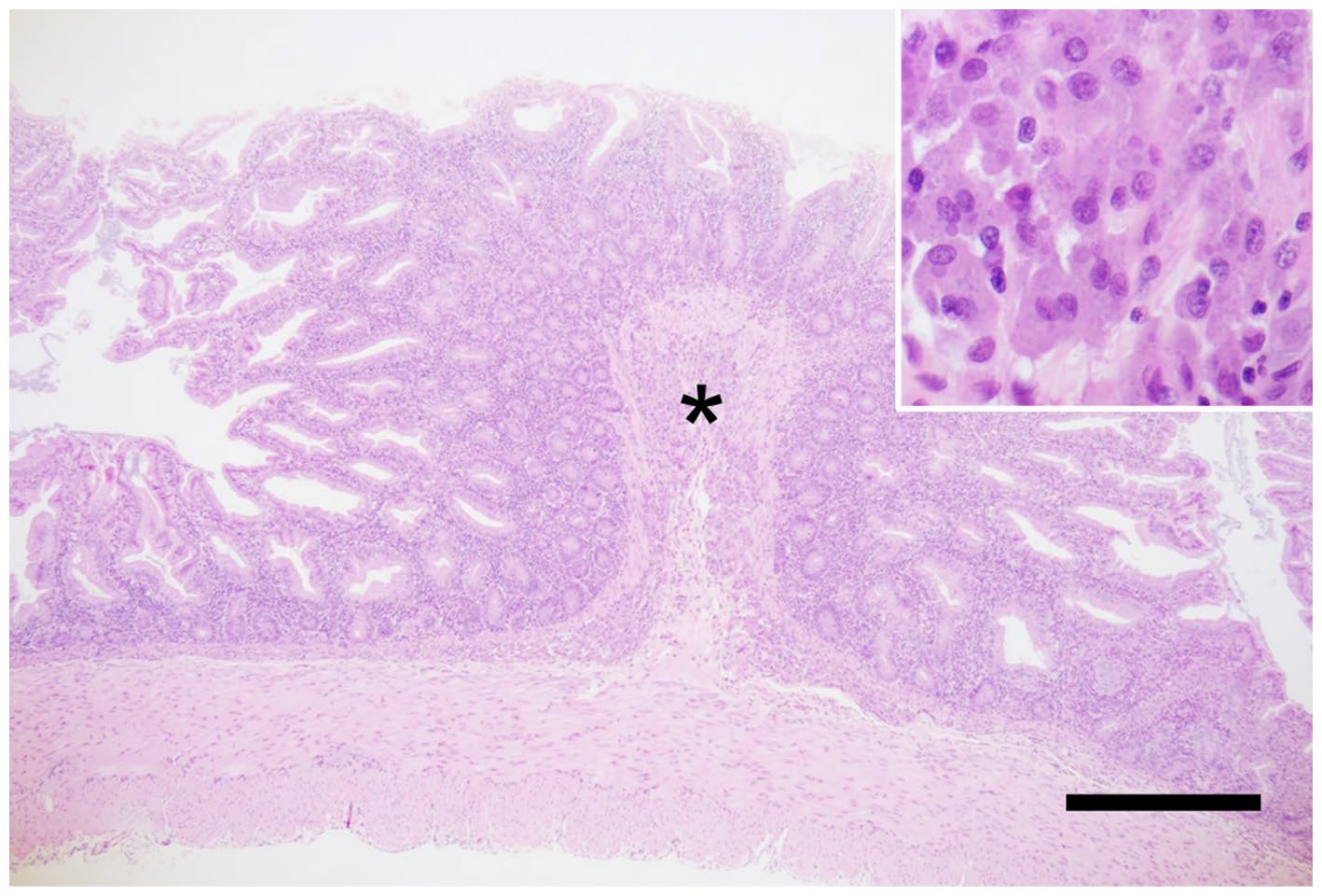
Marmoset wasting syndrome, ileum, hematoxylin and eosin staining. Moderate infiltration of inflammatory cells, predominantly lymphocytes and plasma cells, is observed in the lamina propria. In addition, severe infiltration of macrophages is seen in the submucosa (asterisk), corresponding to the circular folds. The inset shows macrophages with abundant cytoplasm. Scale bar = 1,000 μm.

Gross weight loss, decreased muscle mass, and alopecia are the primary symptoms of MWS. Histopathological examination revealed moderate-to-severe infiltration of the lamina propria of the small intestine by inflammatory cells (predominantly lymphocytes and plasma cells) in all 10 patients. Mild-to-severe macrophage infiltration was observed; however, neutrophil infiltration was infrequent (Figure 1). Histopathological analysis revealed suppurative cholangitis/cholecystitis (six of eight). Bacterial cultures of the suppurative bile were performed from five diseased animals: *Escherichia coli* (4/5), *Enterobacter* spp. (1/5), *Streptococcus* spp. (1/5), and *Neisseria* spp. (1/5).

### 3.3 DDS

The second most common condition was DDS, which showed a distinct age distribution and clinical presentation compared to MWS.

During the study period, 41 necropsied marmosets were diagnosed with DDS, representing 20.6% of all cases. These animals included 22 males and 19 females, with no sex predilection (*p* = 1.0). Their ages ranged from 1.8 to 12.8 years (average: 6.2 ± 2.8 years), and 17/41 (41.5%) animals were aged 3–6 years. The mean body weight at the time of death was 272 ± 40 g (Supplementary Tables S1, S2).

We previously reported the clinical findings in marmosets with DDS at the CIEM (13). DDS is clinically characterized by vomiting (occasionally bile-colored), bloating, weight loss, and hypochloremia. Bilious vomiting, which appears green due to bile, is a hallmark of DDS in marmosets. The DDS was associated with hypokalemia in 10 out of 32 cases (31.2%) and hypochloremia in five out of 32 cases (15.6%) (Table 1).

**Figure 2:**
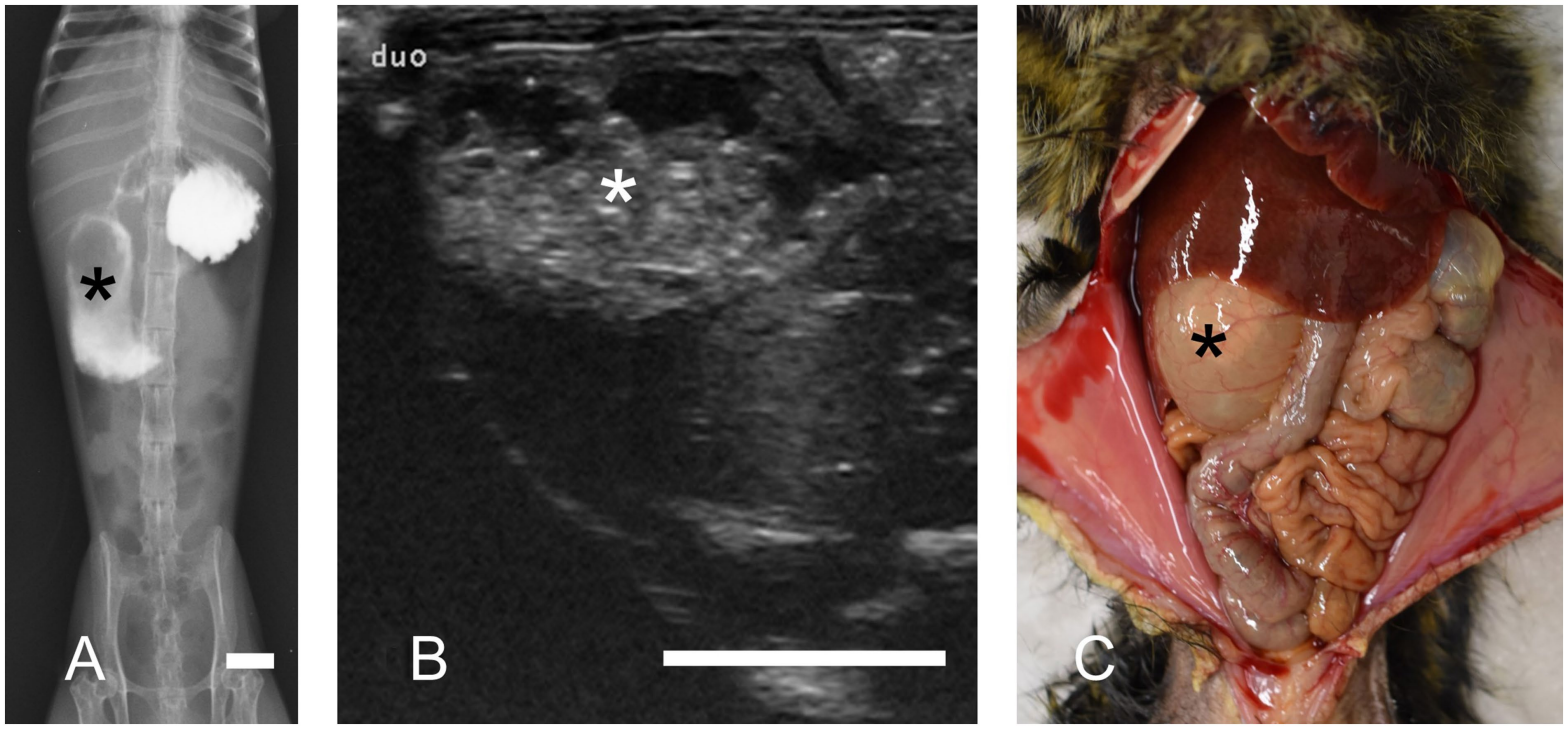
Duodenal dilation syndrome. Marked dilation of the descending duodenum is observed (asterisks). **(A)** Contrast radiography reveals the dilated duodenum. Bar = 10 mm. **(B)** Ultrasonography in transverse section image. Ultrasonography revealed a dilated duodenum on the right side of the abdomen. Bar = 10 mm. **(C)** Necropsy of a marmoset with duodenal dilation syndrome.

Additionally, we reported radiographic and ultrasonographic imaging techniques useful in diagnosing DDS (12). Among them, iodine-based contrast radiography requires the least specialized technique (Figure 2A). However, contrast radiography has certain limitations as the modality may induce diarrhea and can be challenging in severe cases. Contrarily, ultrasonography (Figure 2B) and plain radiography are also effective screening tools. Regardless of the imaging findings, a duodenal dilation of ≥12 mm is considered diagnostic of DDS.

Our previous study described the necropsy findings of DDS (13). They consistently revealed significant dilation of the descending part of the duodenum, which was filled with a mixture of gas and fluid (Figure 2C). Histopathological examination revealed chronic lesions, including chronic peritonitis with connective tissue proliferation between the duodenum and colon, cholangitis/cholecystitis (frequently suppurative), chronic lymphocytic enteritis, and pancreatic ductitis. Severe inflammation is rarely observed unless accompanied by ulceration, making the histopathological features distinctly different from those of MWS. Bacterial cultures of bile samples from 11 cases revealed *Escherichia coli* as the sole isolate in eight, *Enterococcus gallinarum* in one, *Neisseria* spp. in one, and *Escherichia coli* with *Enterococcus faecium* in one case. In contrast, one case demonstrated no bacterial growth.

In this study, aspiration pneumonitis was a complication in 4 diseased cases with DDS. Bacterial cultures were performed in two cases, revealing *Escherichia coli* in one, while the other demonstrated no bacterial growth.

### 3.4 CDAD

Although less frequent in number, CDAD was notable for its acute course and high rate of non-euthanasia deaths, distinguishing it from the other two major diseases.

Nineteen necropsied marmosets were diagnosed with CDAD, accounting for 9.9% of cases during this period. These animals included eight males and 11 females, and their ages ranged from 1.4 to 15.1 years (average: 7.4 ± 3.8 years). No sex predilection was observed (*p* = 1.0). Of the 19 marmosets with CDAD, 6 were euthanized and 13 died without euthanasia, including 8 that did not respond to treatment and 5 that died suddenly. The risk of death without euthanasia (13/19, 68.4%) was significantly higher for the animals with CDAD than for those with the other major diseases (*p* < 0.001). The mean body weight at the time of death was 269 ± 31 g (Supplementary Tables S1, S2).

**Figure 3:**
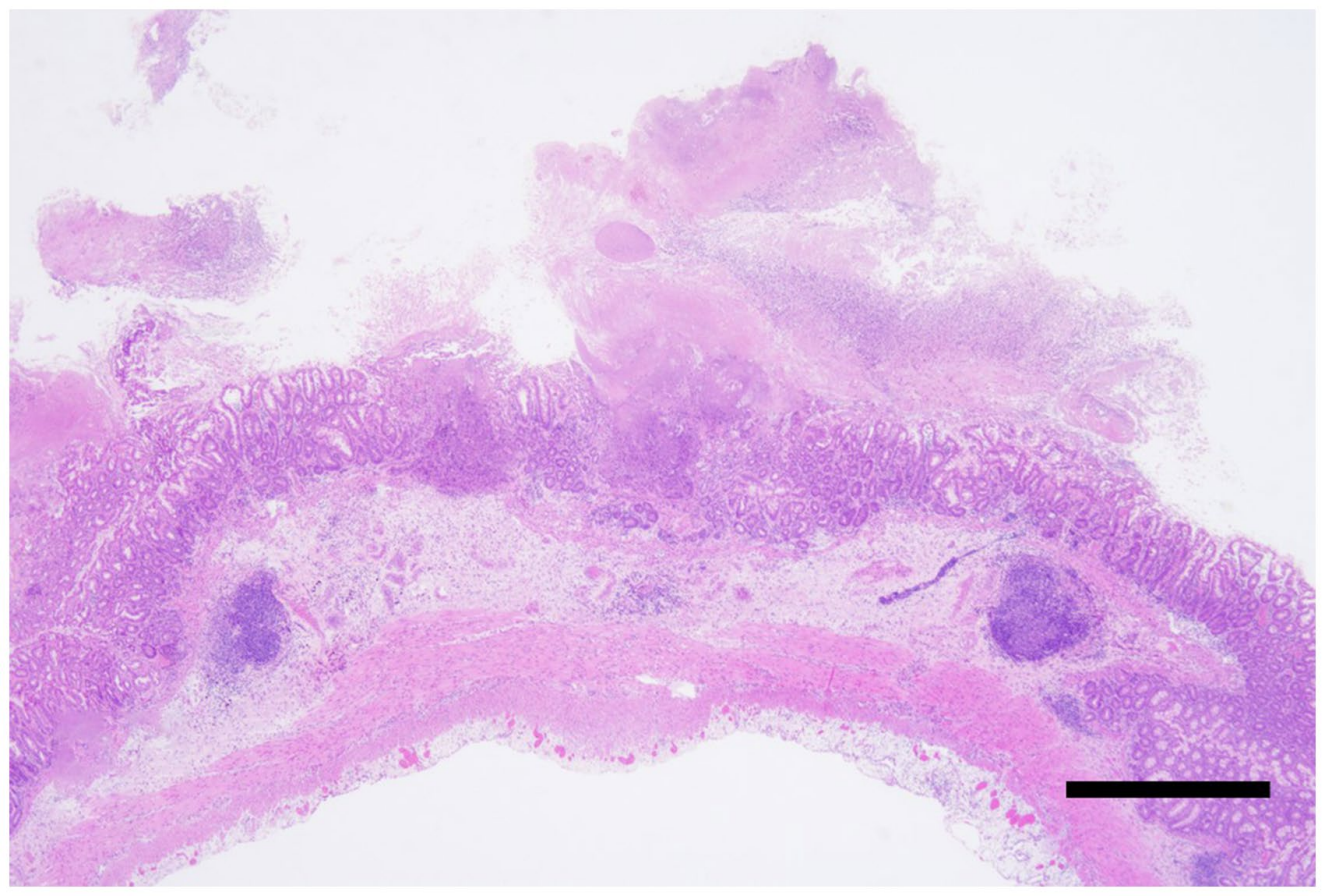
*Clostridioides difficile*-associated colitis, colon, Hematoxylin and eosin staining. Severe necrosis of the intestinal wall with the formation of pseudomembranes. The lesion extends transmurally, reaching the serosa. Bar = 500 μm.

These pathological characteristics are consistent with those of previous reports of CDAD in humans and marmosets. At necropsy, the marmosets showed dilation and congestion of the serosal surface of the large intestine. The colonic mucosa was covered with large amounts of a clear, thick, gelatinous mucoid material called a pseudomembrane. Histologically, pseudomembranous enterocolitis with fibrin is characterized by fibrin aggregates and nuclear debris along the mucosal surface (Figure 3), which was observed in all cases positive for *C. difficile*.

### 3.5 Hemoperitoneum

**Figure 4:**
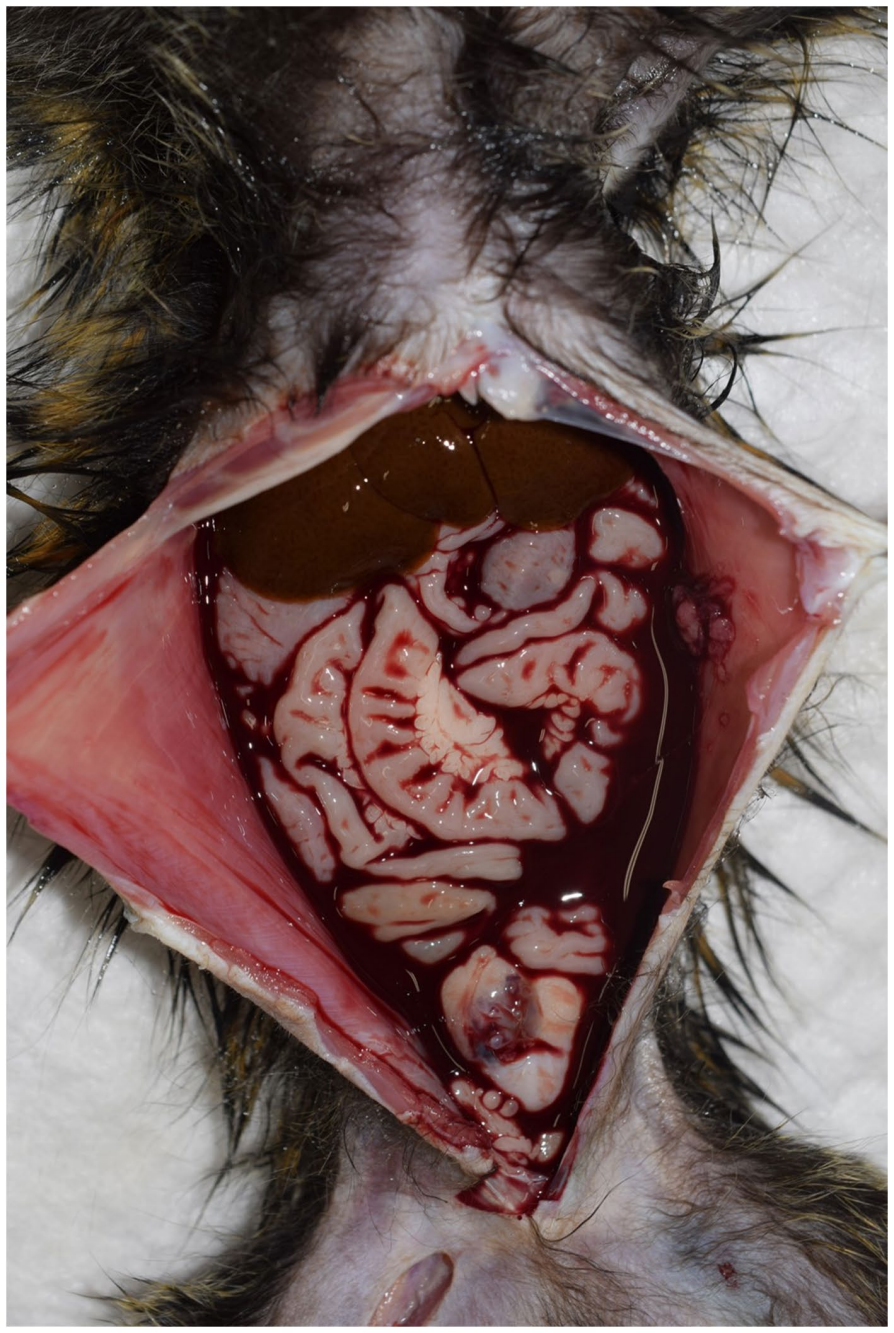
Necropsy of a marmoset with hemoperitoneum. A large amount of hemorrhage is observed within the abdominal cavity. In most cases, it is challenging to identify the exact site of bleeding.

Six necropsied marmosets were diagnosed with hemoperitoneum, accounting for 3.1% of the cases during this period. Hemoperitoneum developed acutely, and all diseased animals died naturally or were euthanized within 24 h after clinical abnormalities were noticed. A large amount of blood was observed in the abdominal cavity during necropsy in all cases (Figure 4). Identifying the bleeding sites was challenging in several cases. However, hepatorrhexis was suspected in one animal, and a hepatic scar with hemosiderin deposition was observed, suggesting a history of recurrent hemorrhage.

### 3.6 Small intestinal adenocarcinoma

**Figure 5:**
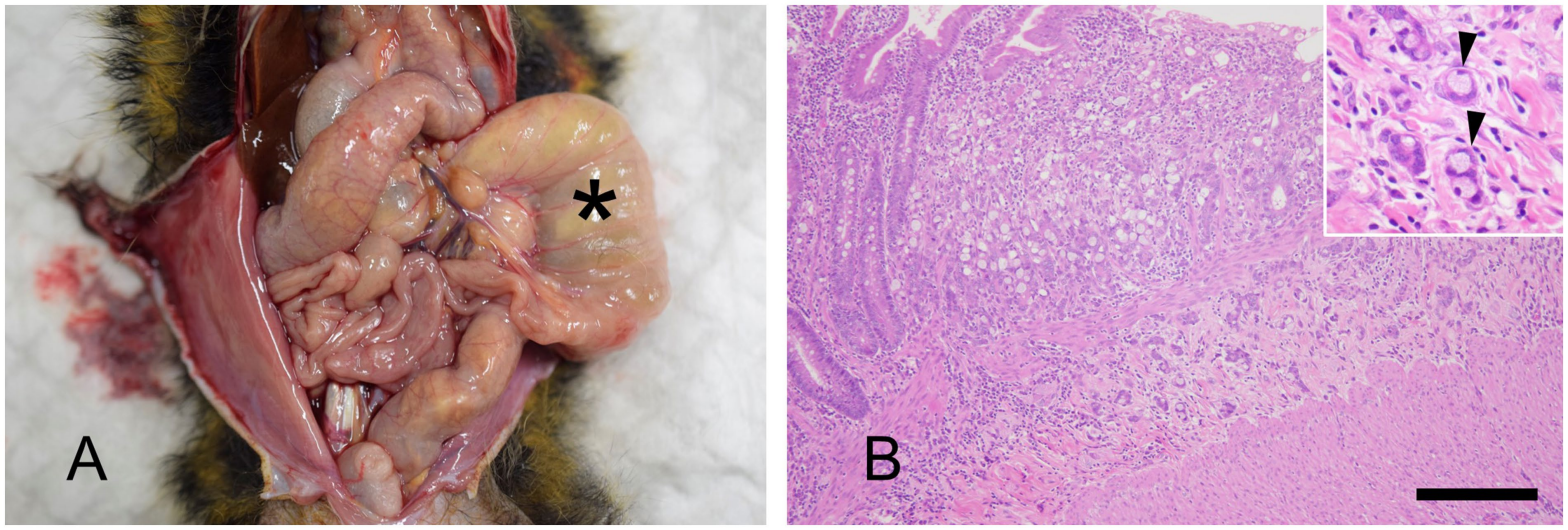
Small intestinal adenocarcinoma. **(A)** Necropsy of a marmoset. Marked dilatation of the small intestine is observed (asterisk). Distinguishing this from duodenal dilation syndrome, which primarily affects the duodenum (see Figure 2), is important. However, in some cases, differentiation between the two diseases can be difficult. A visible mass is not always detected, and histopathological examination of the distal end of the dilated section is crucial for diagnosis. **(B)** Hematoxylin and eosin staining. Invasive proliferation of epithelial tumor cells is observed, extending beyond the muscularis mucosae. The inset highlights signet ring cells (arrowhead). Bar = 500 μm.

Five necropsied marmosets were diagnosed with small intestinal adenocarcinoma, accounting for 2.6% of the cases during this period. We did not observe adenocarcinomas in the large intestine. Adenocarcinomas in marmosets are often not grossly noticeable. The tumor lesion was sclerotic, and the proximal small intestine was dilated (Figure 5A). Distinguishing them from DDS can be challenging. Histologically, most of the neoplastic cells contained intracytoplasmic vacuoles that displaced the nucleus to the periphery (signet ring cell differentiation) (Figure 5B).

### 3.7 Gastrointestinal tract lymphoma

**Figure 6:**
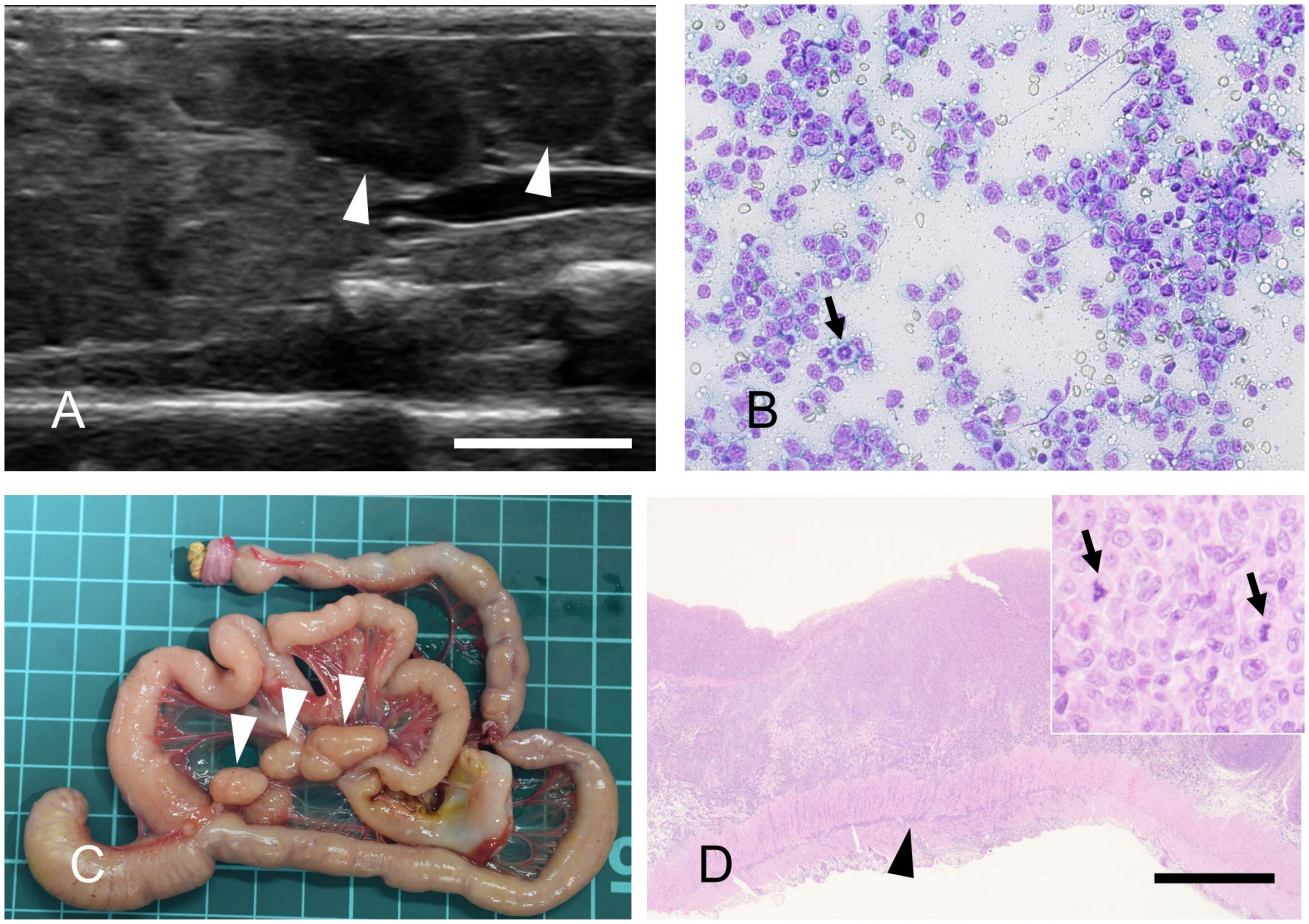
Gastrointestinal lymphoma. **(A)** Abdominal ultrasonography reveals mesenteric lymph nodes exceeding 1 cm in diameter. Bar = 10 mm. **(B)** Fine-needle aspiration, modified Giemsa stain. The proliferation of monomorphic lymphocytes, each with nuclei approximately twice the size of erythrocytes, is observed. A mitotic figure is also occasionally seen (arrow). **(C)** Gross appearance of the gastrointestinal tract at necropsy. Marked enlargement of the mesenteric lymph nodes is observed (arrowheads). The background grid represents a 1 cm scale. **(D)** Hematoxylin and eosin staining. Neoplastic lymphocytes infiltrate transmurally. Arrowhead indicates neoplastic cell invasion into the muscularis layer. Bar = 1,000 μm. Inset: High-magnification image. The neoplastic cells possess large nuclei that are more than twice the diameter of an erythrocyte, with mitotic figures frequently observed (arrows).

Three necropsied marmosets were diagnosed with gastrointestinal tract lymphoma, accounting for 1.6% of cases during this period. The mean age of the affected animals was 8.4 years. Lymph node enlargement can often be detected by abdominal ultrasonography (Figure 6A). In marmosets, lymph nodes greater than 1 cm in diameter frequently indicate the presence of neoplastic lesions. Cytological examination of these nodes enables a less invasive diagnostic approach (Figure 6B). Necropsy revealed masses in the gastrointestinal tract and marked enlargement of the mesenteric lymph nodes (Figure 6C). Histopathological examination frequently reveals transmural infiltration of neoplastic lymphocytes, which are large, with prominent nuclei (Figure 6D).

## 4 Discussion

In this study, MWS, DDS, and CDAD were the leading causes of mortality in a marmoset colony at the CIEM, accounting for approximately 62.0% of all cases. MWS commonly affected older marmosets, with an average age of 9.7 ± 2.3 years. It often led to euthanasia due to chronic weight loss and deterioration. DDS primarily occurred in younger animals with an average age of 6.2 ± 2.8 years and also resulted in euthanasia. CDAD is an acute disease characterized by a high rate of sudden death, and prompt diagnosis and treatment are essential. Understanding the pathogenesis of these diseases and establishing effective prevention and treatment strategies are vital for improving the welfare of marmosets. To the best of our knowledge, this is the first comprehensive study to identify DDS and CDAD as leading causes of mortality in a large, single-center, captive marmoset colony. Previous reports have described these conditions sporadically; however, their relative impact and clinicopathological characteristics have not been systematically investigated. In addition, neoplastic diseases such as small intestinal adenocarcinoma and gastrointestinal lymphoma were observed, reflecting the health problems faced by marmoset colonies, particularly gastrointestinal lesions.

In previous studies, more emphasis has been placed on histopathological findings than on diagnostic labels. However, our study presented results based on diagnoses that were directly related to the causes of death. As shown in Supplementary Table S4 (9–11, 18, 19, 27), investigations from the 1980s primarily highlighted histopathological findings, such as renal interstitial infiltrates and hemolytic anemia. These findings differ from those of the major causes of death identified in our study, making direct comparisons challenging. Nonetheless, the incidence rates of conditions such as small intestinal adenocarcinoma remain relevant and informative (11). The chronic enteritis reported by Tucker (9) may correspond to what we have identified as MWS. Indeed, chronic enteritis was histologically confirmed in all MWS cases in our study.

Moreover, this study identified DDS and CDAD as significant causes of mortality in marmoset colonies. These diseases have not been emphasized as major issues in previous studies. Such discrepancies may be attributed to differences in colony management practices, including breeding plans, facilities, animal sources, or variations in the microbiota of the different colonies (see Supplementary Table S4). Our findings highlight the importance of individualized feeding protocols, early diagnostic interventions, and cautious use of antibiotics, underscoring the need for comprehensive health management strategies tailored to specific colony conditions. The following sections discuss the clinical manifestations, pathology, and veterinary management strategies of each disease. While these findings are based on a single institution, they have broader implications for captive non-human primate research. The disease patterns observed here, particularly the emergence of DDS and CDAD, may reflect underlying vulnerabilities common to marmoset colonies globally, such as dietary stressors, antibiotic exposure, and microbiota disruption. Therefore, our results may inform health management strategies at other institutions housing marmosets or related species.

### 4.1 MWS

MWS is a poorly understood but major disease in captive marmoset colonies characterized by progressive weight loss, muscle atrophy, alopecia, diarrhea, and enteritis (4–6, 26, 28). Its prevalence ranges 28–60% in captive colonies globally (26). Various etiologies, such as food allergies, infectious diseases, and autoimmune conditions, have been proposed, but the exact pathogenesis remains unknown (26). Several studies have proposed that malabsorption due to enteritis may be a primary or secondary contributing factor (5, 10, 29). To date, no curative treatment has been established for patients with MWS. However, some therapeutic approaches are promising. Recent studies have reported that tranexamic acid may reduce gastrointestinal inflammation (30). It has also been shown to improve clinical symptoms. Budesonide, a glucocorticoid with few systemic side effects, increases body weight and serum albumin concentrations in affected marmosets (6). In our study, pancreatin administration yielded satisfactory results in some cases (data not shown). In contrast, the response to prednisolone treatment was inconsistent in our study (data not shown). These findings suggest that targeted therapies may help ameliorate the clinical signs of MWS, although no definitive cure exists, underscoring the need for further research to develop standardized treatment protocols.

A survey conducted between 1989 and 1993 among veterinarians and administrators in North American zoos identified nutritional deficiencies, infectious enteritis, and dietary allergies as the primary causes of MWS (28). More recently, a 2018 study conducted in European zoos highlighted the role of environmental stressors such as the proximity of predator enclosures and inadequate housing as significant contributors to the development of MWS (31). Their study further highlighted the benefits of providing naturalistic environments, including access to trees and nest boxes, to reduce stress and mitigate the risk of MWS. These findings suggest that enhancing environmental enrichment, in addition to dietary improvements, may play an important role in preventing MWS in captive marmosets.

This study’s findings indicate that MWS is associated with significant biochemical and hematological abnormalities. Hypoalbuminemia was observed in 42 of 55 animals, suggesting issues with nutrient absorption or protein synthesis, which is consistent with previous findings on gastrointestinal disease and albumin concentrations in marmosets (26). Elevated total bilirubin in 34 of the 54 animals suggested possible biliary tract dysfunction, which was further supported by the presence of cholangitis/cholecystitis on histopathological examination. The detection of bacterial infections further implicates infectious agents in these biliary issues. These results emphasize the systemic nature of MWS and the need for early diagnosis and targeted treatment to improve outcomes in affected marmosets.

Enhancing nutritional management is paramount to mitigate the impact of MWS. Given the potential link between MWS and malnutrition or malabsorption, revision of the dietary regimen is warranted. Providing a high-protein, nutrient-dense diet supplemented with digestive enzymes and probiotics may improve the overall health and absorption efficiency of marmosets. Stress management also plays a critical role in the prevention of MWS. Therefore, enrichment strategies that stabilize group dynamics and reduce environmental stressors should be prioritized. Moreover, implementing early diagnostic measures, such as regular blood tests and body weight monitoring, may facilitate the early detection and treatment of MWS and improve the prognosis.

### 4.2 DDS

In 2020, DDS was reported as a novel gastrointestinal disorder in common marmosets (13) and has since been identified in colonies globally (14, 15, 32). Despite their prevalence, preventive measures and curative treatments remain underdeveloped, highlighting the need for tailored management strategies. Dietary modifications are critical, with smaller and more frequent feedings recommended to reduce digestive stress, while high-fiber foods such as fruits should be avoided. In addition, the use of liquid diets, such as Maybalance 2.0 (Meiji Co., Ltd., Tokyo, Japan), as a supplementary measure for nutritional support is effective. Pharmacological treatments, including antacids such as famotidine for managing duodenal ulcers and prokinetics such as metoclopramide (Primperan; Astellas Pharma Inc., Tokyo, Japan), may help address gastrointestinal dysfunction, although further research is needed to ascertain their efficacy. In severe cases, gastric decompression through catheterization is recommended to alleviate distension, and dimethicone (Gascon Drop Oral Solution 2%; Kissei Pharmaceutical Co., Ltd., Nagano, Japan) can be used to manage gas accumulation. Surgical interventions, such as partial resection of the stomach or duodenum with anastomosis, may be considered but require specialized expertise and careful risk assessment. In principle, anesthesia should be avoided; however, premedication with maropitant (Cerenia; Zoetis Japan, Tokyo, Japan) and intubation should be performed, if necessary. For patients with moderate-to-severe symptoms, treatment should include symptomatic care, such as warming for hypothermia and subcutaneous infusion for electrolyte correction. Correcting hypokalemia and hypochloremia based on blood test results is particularly important. Ongoing research on the pathophysiology of DDS, including gut microbiota analysis, histopathological examination, and imaging diagnostics, is expected to advance veterinary care for this complex condition.

### 4.3 CDAD

*C. difficile* is an obligate, anaerobic, spore-forming, Gram-positive rod that inhabits the intestinal tracts of humans and various mammals and is a major cause of nosocomial infections in humans (33, 34). Several recent studies have documented CDAD in New World monkeys, including common marmosets (3, 16, 17). In our study, *C. difficile* was detected in the feces of healthy marmosets using bacterial antigens, indicating that *C. difficile* is a commensal bacterium under normal conditions. However, individuals housed in proximity to the affected animals frequently develop CDAD, suggesting horizontal transmission. The administration of antimicrobial agents, particularly new quinolones and beta-lactams, is a significant risk factor because these agents disrupt the gut microbiota and allow *C. difficile* overgrowth and enteritis (33).

Clinically, CDAD in marmosets is characterized by mucous stools, severe weight loss (often exceeding 30 g within days), reduced stool volume, and decreased activity. Early detection is critical but challenging, as 68.4% of cases in our study were identified postmortem, highlighting the rapid progression of the disease. The high proportion of non-euthanasia deaths in CDAD cases likely reflects the rapid disease progression and difficulty in early clinical recognition. In some instances, affected animals exhibit no apparent fecal abnormalities (e.g., absence of diarrhea or mucus), especially in group-housed settings where only the feces of unaffected individuals may be observed. Consequently, overt signs may be missed, leading to delayed intervention. These findings underscore the importance of monitoring subtle behavioral changes, such as reduced activity or appetite, to facilitate earlier detection and improve clinical outcomes. When clinical signs such as mucous stools or significant weight loss are observed, CDAD diagnosis should be confirmed using a human CDAD detection kit, such as C Diff Quick Chek Complete. Treatment with metronidazole or vancomycin is effective; however, recurrence is common and requires close monitoring. Metronidazole is typically administered subcutaneously at a dose of 20 mg/kg/day once or twice daily (sid or bid). In contrast, vancomycin is administered orally at a dose of 25 mg/kg two to three times daily (bid or tid). For severe cases, both drugs may be used in combination with the appropriate dosing regimens. However, the widespread use of these antibiotics raises concerns about the emergence of antimicrobial-resistant strains in other bacterial species within the gut microbiota. This underscores the importance of antimicrobial stewardship in minimizing the development of resistance.

Preventing CDAD relies on the judicious use of antibiotics because their administration is strongly associated with disease onset. Probiotic supplementation with antibiotics may help preserve the intestinal microbiota and reduce the risk of CDAD. Fecal transplantation has also been shown to be an effective treatment strategy for *C. difficile* infection in marmosets (16). Early diagnosis through regular fecal screening and prompt treatment is essential for effective management. In addition, implementing robust infection control measures such as isolating affected individuals and enhancing environmental sanitation is critical for minimizing the spread of the disease within colonies. Addressing the risk of antimicrobial resistance by carefully monitoring antibiotic use and exploring alternative therapies will play a pivotal role in sustainable disease management. In human medicine, antimicrobial stewardship programs have been implemented to minimize the emergence of resistant organisms and reduce the incidence of CDAD (35). A comparable strategy should be adopted in captive marmoset colonies by developing colony-specific antibiotic use guidelines tailored to local microbial ecology and clinical risk factors.

### 4.4 Hemoperitoneum

Hemoperitoneum is the leading cause of death. Hemoperitoneum has rarely been reported in marmosets (36). In several cases, the site of bleeding is unknown. Premortem diagnosis can be made by aspirating blood from the abdominal cavity under ultrasound guidance. Blood transfusion (24) and hemostasis through exploratory laparotomy have shown potential efficacy in marmosets with early-stage hemoperitoneum. Unfortunately, we did not encounter any cases in which marmosets with hemoperitoneum were successfully treated.

In gastrointestinal tract lymphomas, thickening of the small intestinal wall and enlargement of the mesenteric lymph nodes may occasionally be palpated. In several cases, abdominal ultrasonography reveals masses in the gastrointestinal tract or mesenteric lymph nodes. Fine-needle aspiration of these masses under ultrasound guidance can facilitate the diagnosis of gastrointestinal lymphoma. In addition, neoplastic lymphocytes may be detected in peripheral blood, superficial lymph nodes, or thoracoabdominal effusion, as the disease progresses.

### 4.5 Tumors

The incidence of tumors in the CIEM was 4.2%, all of which were small intestinal cancers or gastrointestinal lymphomas. Tumor development in marmosets has been reported, with small intestinal cancer being the most common neoplasm. This was also the case at the CIEM, where it showed the highest incidence among tumors. In agreement with a report by Miller *et al.* (11), small intestinal cancer was characterized by the presence of signet ring cells. Gastrointestinal tract lymphoma is also a common disease in marmosets (10). Differential diagnoses included other conditions characterized by progressive weight loss or malabsorption, such as MWS, and other neoplastic conditions, including small intestinal adenocarcinoma. To date, no established treatment has been reported for these tumors.

### 4.6 Limitations

This study is retrospective, limiting causal inference. Data were collected from a single institution, restricting the generalizability of our findings. Small sample sizes for certain diseases reduced the statistical power. Advanced diagnostic techniques such as microbiome analysis and computed tomography imaging were not routinely performed, potentially missing subclinical conditions. In addition, the predominance of euthanized cases may have introduced a bias toward severe conditions.

## 5 Conclusion

A multifaceted approach is essential to achieve a comprehensive understanding of disease patterns in marmoset colonies. Factors such as diet, genetic predispositions, gut microbiota, and experimental history are likely to influence disease development and require thorough investigation to establish effective prevention and treatment strategies. Addressing environmental factors, optimizing dietary management, and implementing stress-reduction measures are critical components of health management. Regular necropsies and histopathological examinations should be integrated into these efforts. By combining these strategies, marmoset welfare can be enhanced, research outcomes improved, and the challenge of maintaining healthy colonies can be effectively addressed. These findings not only provide new insights into major causes of mortality in captive marmosets but also emphasize the need for institution-specific veterinary guidelines and further multicenter investigations.

## Data Availability

The raw data supporting the conclusions of this article will be made available by the authors, without undue reservation.
